# Auditory processing assessment in children with obstructive sleep apnea syndrome

**DOI:** 10.1016/S1808-8694(15)30963-0

**Published:** 2015-10-19

**Authors:** Karin Neves Ziliotto, Maria Francisca Colella dos Santos, Valeria G. Monteiro, Márcia Pradella-Hallinan, Gustavo A. Moreira, Liliane Desgualdo Pereira, Luc L.M. Weckx, Reginaldo Raimundo Fujita, Gilberto Ulson Pizarro

**Affiliations:** a Doctor in the Science of Human Communication Disturbances Unit at UNIFESP/EPM, Phonoaudiologist; b Doctor in the Science of Human Communication Disturbances Unit at UNIFESP/EPM, Lecturer of the Phonoaudiology course/Medical Science College - FCM/UNICAMP; c Medical ENT Specialist and Lecturer of Pediatric Otorhinolaryngology - UNIFESP/EPM; d Doctor, Lecturer on Sleep Medicine and Biology. Psychobiology Department at UNIFESP/EPM; e Doctor, Lecturer on Sleep Medicine and Biology. Psychobiology Department at UNIFESP/EPM; f Doctor in the Science of Human Communication Disturbances at UNIFESP/EPM, Associate Medical Professor in Auditory Disturbances at the Phonoaudiology Department at UNIFESP/EPM; g Associate Professor, Full Professor of Pediatric Otorhinolaryngology at UNIFESP/EPM; h Medical Doctor graduate from Sao Paulo Federal University - UNIFESP, Head of the Pediatric Otorhinolaryngology Clinic - UNIFESP; i Master in Otorhinolaryngology at Sao Paulo Federal University - UNIFESP, Medical ENT specialist. Sao Paulo Federal University - Paulista Medical College

**Keywords:** Obstructive sleep apnea, audiology, hearing tests

## Abstract

**Introduction:**

The obstructive sleep apnea syndrome (OSAS) is a respiratory disorder that occurs during sleep and it is relatively common in children.

**Aim:**

The goal of this paper is to verify if there is a relationship between the obstructive sleep apnea syndrome (OSAS) and auditory processing.

**Methods:**

In order to do that, three groups of children ranging in age from 5 to 11 were studied, including a normal group. Twenty subjects who made up the study group were submitted to ear, nose and throat (ENT) exams and to polysomnography (PSG), and were divided in two groups: GROUP I (RO) comprised of 10 children who presented oral breathing and displayed normal PSG, and GROUP II (SAS) comprised of 10 children who presented oral breathing and displayed abnormal PSG. Their performance was compared to the performance of the third group - GROUP III (REN) comprised of 10 children who did not refer ENT difficulties. All the subjects completed a basic audiometric assessment as well as an auditory processing diagnosis.

**Results:**

The analyses of the results revealed a statistically significant difference in ENT exams related to the turbinate and the palatine tonsils. Group II presented a higher incidence of turbinate hypertrophy levels II and III (p < 0.001) and palatine tonsils hypertrophy grades III and IV (p 0.007). Regarding the auditory processing assessment, a statistically significant difference (p < 0.001) was obtained in the dichotic digits test. Group II performed worse than group III. Also, for the non-verbal sequence memory test, Group II obtained a worse result (p < 0.022) than Group I.

**Conclusion:**

Subjects with OSAS obtained worse results in auditory processing tests.

## INTRODUCTION

Obstructive sleep apnea/hypopnea syndrome (SAHS) has been described as a relatively common entity in children. SAHS in children is a sleep breathing disorder characterized by partial or full upper airway obstruction that interferes in ventilation and the normal sleep pattern^1^, ^2^. The prevalence of SAHS according to various studies varies between 1% and 3% in children aged 2 to 18, with no gender predominance^2^.

Structural changes such as pharyngeal and palatine tonsil hypertrophy are the most common etiological factor of obstructive apnea^1^, ^2^, ^3^, ^4^. Other causes of SAHS include obesity, craniofacial anomalies, neuromuscular diseases, hypothyroidism and genetic syndromes. These causes are, however, less common and will not be dealt with in this paper. Surgical treatment to remove the hypertrophied pharyngeal and palatine tonsils is the treatment for most children with SAHS^2^, ^3^, ^5^, ^6^, ^7^.

Symptoms in children with SAHS include snoring, breathing difficulty and/or breathing pauses during sleep, excessive sweating and enuresis. Day time symptoms may include oral breathing, headaches and excessive sleepiness^1^, ^2^, ^6^.

The polysomnographic recording is essential for the diagnosis of SAHS, together with the clinical history, the physical examination and laboratory tests^1^.

SAHS may cause pulmonary hypertension, anomalies in gas exchanges, cardiac and respiratory changes, low growth development, sleep fragmentation, behavioral and neurocognitive changes^4^, ^6^, ^8^ (attention deficit, hyperactivity, learning difficulties, memory loss and intelligence deficits).

Behavioral and neurocognitive changes and impaired school performance have been frequently described in children and adults with SAHS. No studies have yet established a correlation between assessments of peripheral and central hearing function and SAHS in children.

It is known that hearing in a normal subject is not restricted to the peripheral sensorial level, but also involves central auditory pathways, the cerebral cortex and efferent auditory pathways. The clinical presentation of patients with auditive processing impairment include attention deficit, speech and associated language disturbances, difficulty in understanding speech in noisy or acoustically unfavorable environments and impaired learning at school^9^.

Thus, the aim of this study is to assess if there is any correlation between SAHS and changes in auditory processing.

## CASES AND METHODS

This study was conducted as a partnership between the Pediatric Otorhinolaryngology Department and the Auditory Disturbances Unit of the Federal University of Sao Paulo. The study design was evaluated and approved by the Research Ethics Committee of the Sao Paulo Federal University / Sao Paulo Hospital, protocol number 0448/02. Oral and written information was given to all patients concerning the objectives and procedures of the study. Patients signed a free and informed consent form after agreeing to participate in the study.

Between March 2001 and July 2002, 30 patients aged from 5 to 11 were evaluated and divided into three groups:

• GROUP I - 10 oral breathers with a normal polysomnographic recording - named the OB group (Oral Breather group).
• GROUP II - 10 oral breathers with an altered polysomnographic recording - named the SAHS Group (group with Obstructive Sleep Apnea/Hypopnea Syndrome).
• GROUP III - 10 nasal breathers, with no otorhinolaryngological, hearing and/or school complaints - named the NB Group (Nasal Breather group).

All patients in this study had a baseline normal audiological assessment^10^, ^11^ which included pure tone audiometry, logoaudiometry and imitanciometry. The normal value was audition present at levels below 25dBNA in all pure tone audiometry evaluated sound frequencies^10^, ^11^ and a normal tympanic curve (type A) with acoustic reflections in contra-lateral mode at imitanciometry sound frequencies of 500Hz, 1000Hz, 2000Hz and 4000Hz. All patients underwent auditory processing screening following the basic audiological assessment.

Table 1 presents the age distribution of the sample, and Table 2 presents the groups according to gender.

**Table 1:** Average age (years) in groups I (OB), II (SAHS) and III (NB).

Age	Group I	Group II	Group III
Average	8,1	7,6	7,5

**Table 2:** Gender distribution in groups I, II and III.

Gender		Group I	Grup II	Grup III
Male	N	6	7	5
	%	60%	70%	50%

Female	N	4	3	5
	%	40%	30%	50%

Children with facial malformation, genetic syndromes and neurological diseases were excluded from the study due to possible central nervous system involvement and peripheral hearing loss. Children with a polysomnographic diagnosis of primary snoring were also excluded.

Patients in groups I and II underwent otorhinolaryngological screening and polysomnography, as follows:

### 1) Otorhinolaryngological Screening

Every patient in groups I (OB) and II (SAHS) underwent otorhinolaryngological screening done by a single health professional, results which were not confirmed by another ENT medical specialist. The examination included a physical exam, otoscopy, anterior rhinoscopy, oroscopy and nasofibrolaryngoscopy using a 3.2 diameter flexible optical fiber to assess the size of the pharyngeal and palatine tonsils.

Anterior rhinoscopy allowed an assessment of the nasal turbinates which were classified according to the following parameters12:

• No edema: Normal inferior nasal turbinate.
• Grade I nasal turbinate hypertrophy: inferior turbinate is 25% of the nasal fossa.
• Grade II nasal turbinate hypertrophy: inferior turbinate is 50% of the nasal fossa.
• Grade III nasal turbinate hypertrophy: inferior turbinate is 75% or more of the nasal fossa.

The size of the pharyngeal tonsil was estimated based on the percentage of the posterior area of the inner nose occupied by adenoid tissue^12^ and classified as:

• Obstructive: the tonsils occupy more than 70% of the inner nose;
• Non-Obstructive: the tonsils occupy less than 70% of the inner nose.

Palatine tonsils were classified according to Brodsky's classification system13:

• Grade I: palatine tonsils occupy less than 25% of the oropharynx.
• Grade II: palatine tonsils occupy more than 25% and less than 50 % of the oropharynx.
• Grade III: palatine tonsils occupy more than 50% and less than 75 % of the oropharynx.
• Grade IV: palatine tonsils occupy more than 75% of the oropharynx.

### 2) Polysomnographic Recording

Group I (OB) and II (SAHS) children underwent polysomnography at the Sleep Institute of the Federal University of Sao Paulo. Computerized electrophysiological and cardiorespiratory parameters were used - electroencephalogram, submentonian and tibial electromyography, right and left electro-oculogram, oronasal air flow, thoracical and abdominal movement, microphone (snoring), oxy-hemoglobin saturation (SaO2) and position on the bed. Results were analyzed using the American Thoracic Society Consensus^14^ guidelines that recommends the following parameters: respiratory disturbance index (RDI - the number of apnea/hypopnea events per hour), the mean oxygen saturation during REM and NREM sleep, minimum oxyhemoglobin desaturation (nadir SaO2), sleep efficiency, total sleep time (TST), REM sleep, slow wave sleep, arousal index, duration of apnea and obstructive hypoventilation.

Only the three most important factors (RDI, Nadir and the mean oxygen saturation) were considered to simplify polysomnographic analysis.

### 3) Auditory Processing Assessment

All patients in this study underwent auditory processing screening done by two phonoaudiologists and included the following tests: the sound localization test (SL), the auditory memory test for verbal sounds (MVS) and non-verbal sounds in sequence (MNVS) and the dichotic digits test (DDT). The GSI 61 two-channel audiometer and a Panasonic compact-disc were used for these tests.

A bell was used for the sound localization test (SL). No visual cues were given as this instrument was hit in five positions relative to the patient's head: in front, above, behind, to the left and to the right. Instructions were given by demonstration. The required response was to locate the source of the sound. This test provides information about the physiological auditory mechanism of binaural interaction. Sound localization is the auditory ability evaluated in this test^9^.

The memory test for verbal and non-verbal sounds in sequence provides information about the physiological auditory mechanism of temporal processing. The auditory ability assessed here is temporal ordering^9^. The auditory memory test for verbal sounds was applied using 3 syllables in children aged 5 and 4 syllables in children aged 6 and above^9^, ^15^. Patients were initially asked to repeat each syllable independently. Then they were instructed to repeat verbally three different sequences with three or four syllables each in the same order as presented, with no visual cues.

Musical instruments were used for the non-verbal sound auditory test presented in three different sequences. Patients were asked to point to musical instruments according to the order in which they were played. Instruction was given by demonstration.

The dichotic digits test provides information about the physiological mechanism of dichotic listening of verbal sounds. In the Portuguese version the test list contains 80 digits or 20 items, each item containing 4 digits numbered from 1 to 9, representing Portuguese language disyllables. Two overlapped digits were presented to each ear simultaneously, 16 applied in the binaural integration stage, in which patients were instructed to repeat aloud the four numbers presented to both ears, regardless of the order of presentation. If patients correctly identified the digits, no report was written on the score sheet. However, an error such as omission or substitution was reported on the score sheet with a line over the corresponding digit. Each digit incorrectly identified was scored as a 1.25% error. The percentage of errors in the right and left ears was thus calculated^16^. The dichotic digits test gives an assessment of the figure-ground auditory ability for verbal sounds by means of a binaural integration task. This test was conducted in an acoustic chamber using headphones and compact disc recorded material.

Statistical analysis was done with non-parametric tests due to the small samples, and included the Kruskal-Wallis test and the Mann-Whitney test. Confidence intervals for a more complete descriptive quantitative analysis were also used, at a 5% significance level.

## RESULTS

This study included 30 children (18 males and 12 females) and the mean age was 7.7 years (Tables 1 and 2).

### Otorhinolaryngological screening results

There was a statistically significant difference between the results of otorhinolaryngological screening of nasal turbinates and palatine tonsils. Group I (OB) had a higher number of patients with grade I nasal turbinate hypertrophy. Group II (SAHS) had a higher number of patients with grade II and III nasal turbinate hypertrophy (Tables 3 and 4). Group II (SAHS) had a higher number of patients with grade III and IV palatine tonsil hypertrophy (Table 5).

**Table 3:** Results of anterior rhinoscopy in groups I (OB) and II (SAHS).

	Group I		Group II	
	N	%	N	%
No turbinate edema	3	30	2	20
Grade I nasal turbinate hypertrophy	7	70	0	0
Grade II and III nasal turbinate hypertrophy	0	0	8	80

**Table 4:** Statistical analysis (p-values) of anterior rhinoscopy results in groups I (OB) and II (SAHS).

Anterior rhinoscopy		Turbinates with no edema	Grade I
Group I	Grade I nasal turbinate hypertrophy	0,074	-
	Grade II and III nasal turbinate hypertrophy	0,060	0,001*

Group II	Grade I nasal turbinate hypertrophy	0,136	-
	Grade II and III nasal turbinate hypertrophy	0,007	< 0,001*

**Table 5:** Results of otorhinolaryngological evaluation of the palatine tonsil and pharyngeal tonsil in groups I (OB) and II (SAHS).

Palatine tonsil	Group I		Group II	
	N	%	N	%
Grade I and II	7	70	2	20
Grade III and IV	3	30	8	80
p-value	0,074		0,007*	

Pharyngeal tonsil	Group I		Group II	

	N	%	N	%
Obstructive	4	40	7	70
Non-Obstructive	6	60	3	30
p-value	0,371		0,074	

(*) statistically significant difference (p< 0.05)

There were no statistically significant differences in pharyngeal tonsil size in groups I and II (Table 5).

### Polysomnographic recording results

All group I (OB) patients had polysomnographic recordings within normal limits. Sixty percent of group II (SAHS) patients had mild changes in polysomnographic recordings and 40% of patients in this group had moderate or severe findings in polysomnographic recordings. The average apnea/hypopnea index (number of events per hour) was 6.02 and the mean was 4.7 in group II (SAS).

### Auditory processing results

Statistical analysis demonstrated a significant difference in auditory processing tests when comparing the auditory behavior of patients in the three groups for sequential non-verbal memory tests and the dichotic digits test (Tables 6 and 7).

**Table 6:** Results of auditory processing tests (in percentage of correctness in groups I (OB), II (SAHS) e III (NB).

		SL			MVS			MNVS			DDT	
	G.I	G.II	G.III	G.I	G.II	G.III	G.I	G.II	G.III	G.I	G.II	G.III
Average	100	96	94	76,64	69,96	86,65	83,32	63,18	79,96	88,99	78,26	95,15
Mean	100	100	100	83	67	100	100	67	67	94,5	76,87	95
Minimal value	100	80	80	0	0	66,6	0	33	66	60	60	79
Maximal value	100	100	100	100	100	100	100	100	100	100	100	100

Legend SL: Sound localization test

MNVS: Memory test for non-verbal sounds in sequence

MVS: Memory test for verbal sounds in sequence

DDT: dichotic digits test

**Table 7:** Results of statistical tests (p-values) comparing auditory processing tests in groups I (OB), II (SAHS) and III (NB).

	SL	MVS	MNVS	DDT
I x II x III	0,197	0,483	0,042 *	< 0,001 *
I x II			0,022 *	0,025
I x III			0,105	0,272
II x III			0,166	< 0,001 *

Legend SL: Sound localization test

MVS: Memory test for verbal sounds in sequence

MNVS: Memory test for non-verbal sounds in sequence

DDT: dichotic digits test

(*) statistically significant difference (p< 0.05)

Statistical analysis showed no significant difference in performance between groups I, II and III in sound localization auditory processing tests and memory tests for verbal sounds in sequence (Tables 6 and 7).

There was a statistically significant mean difference between groups in the dichotic digits test, where group II (SAHS) had a weaker performance compared to group III (NB). There was a statistically significant difference in the memory tests for non-verbal sound in sequence between the groups; group II (SAHS) showed a weaker performance compared to group I (OB). These data are in Table 7.

## DISCUSSION

Obstructive sleep apnea/hypopnea syndrome is increasingly being diagnosed in the pediatric population. Patients present with partial or total upper airway obstruction leading to intermittent interruption of normal ventilation during sleep. Three main variables in the pathophysiology of SAHS determine upper airway collapse and obstruction^17^:

A) Anatomical upper airway changes.
B) Decreased pharyngeal dilator muscle activity.
C) Negative pressure on upper airways generated by the thorax.

A statistically significant difference in anterior rhinoscopy was observed between groups I (OB) and II (SAHS); there were more patients with grades II and III nasal turbinate hypertrophy in group II (SAHS) and more patients with grade I turbinate hypertrophy in group I (OB).

All oral breathers with normal polysomnographic recordings (group I - OB) or with an altered polysomnography (group II - SAHS) had hypertrophic turbinates and/or pharyngeal and palatine tonsils on the otorhinolaryngological examination.

Anterior rhinoscopy showed that 70% of patients in group I (OB) had grade I nasal turbinate hypertrophy whereas 80% of patients in group II (SAHS) had grade II and III nasal turbinate hypertrophy. There was a statistically significant predominance of patients with grade I nasal turbinate hypertrophy in group I (OB) and a statistically significant predominance of patients with grade II and III nasal turbinate hypertrophy in group II (SAHS), which also had more patients with obstructive pharyngeal tonsillar hypertrophy (80% of patients). In group I (OB) 60% of patients had obstructive pharyngeal tonsillar hypertrophy, although this was not statistically significant. There were more patients in group II (SAHS) with statistically significant oropharyngeal obstruction (80% with grade III and IV palatine tonsillar hypertrophy), compared to group I in which 40% of patients had these grades of obstruction.

There were more patients with advanced grades of obstruction in group II (SAHS) in the three otorhinolaryngological parameters assessed. Various authors have stated that pharyngeal and palatine tonsillar hypertrophy is associated with obstructive sleep apnea/hypopnea syndrome^1^, ^2^, ^3^, ^4^, as seen also in this study. Furthermore, nasal turbinate hypertrophy was an important factor related to pharyngeal and palatine tonsillar hypertrophy.

Central auditory processing refers to mechanisms and processes that take place in the auditory system and that are responsible for the following behavioral phenomena^18^:

• Sound localization and lateralization;
• Auditory discrimination;
• Recognition of sound patterns;
• Time aspects of audition (temporal resolution, temporal masking, temporal integration, temporal ordering);
• Reduction in auditory performance in the presence of degraded acoustic signals;
• Reduction in auditory performance in the presence of competing acoustic signals.

These mechanisms and processes are applicable to verbal and non-verbal stimuli and may affect different portions of the brain, including speech and language areas.

Auditory Processing Disorder (APD) has been defined as a loss in one or more of the aforementioned areas.

In this study the following auditory specific tests were used to assess auditory processing: sound localization test, memory test for verbal and non-verbal sounds in sequence and the dichotic digits test. Auditory physiological mechanisms of binaural interaction, temporal processing and dichotic listening for verbal sounds were analyzed based on the performance in these specific auditory tests.

There was a statistically significant difference in the dichotic digits test in different groups; impaired performance was seen in group II (SAHS) compared to group III (NB). A further finding was a statistically significant difference in the memory test for non-verbal sounds in sequence with group II (SAHS) underperforming compared to group I (OB).

Dichotic listening tests (dichotic digits test) and temporal processing tests (memory test for non-verbal sounds in sequence) are recognized as important tools to define disturbances in auditory processing^19^. They are also sensitive to changes in the brain stem, brain cortex injuries and corpus callosum lesions^20^, ^21^.

Consequences of SAHS include significant behavioral disturbances, learning disabilities, attention deficit and hyperactivity, impaired memory and difficulties at school, among others^22^, ^23^, ^24^. Mechanisms by which sleep disturbances contribute to these behavioral changes in SAHS patients are still unclear. Some studies have stated that sleep fragmentation and intermittent hypoxia are significant factors. Intermittent hypoxia seems to cause prefrontal and hippocampus loss of neurons and resulting difficulties in tasks involving spatial memory. Furthermore, experimental studies have demonstrated that neuronal susceptibility to period of hypoxia during sleep coincides with ages when the prevalence of SAHS reaches its peak in children. An experimental study in rats showed significant losses in acquisition and retention in three-dimensional tasks. Male rats exposed to intermittent hypoxia during sleep also had increased motor activity. Another experimental study using rats revealed that medial temporal lobe structures are particularly susceptible to hypoxia^25^. Therefore, late diagnosis and treatment of SAHS can affect multiple organs and systems, and may lead to definitive neuronal disturbances and limits to full neurocognitive development^8^, ^24^, ^26^.

Our study showed that group II (SAHS), which included children with obstructive sleep apnea/hypopnea syndrome, revealed auditory processing disturbances that may be considered as a further presentation of SAHS. Auditory processing disturbances may result from the same mechanisms that cause neurocognitive changes in general.

Preliminary data from this study suggest that it is important to consider auditory processing assessment in the multidisciplinary setting used to clinically evaluate these children - oral breathers with or without changes in polysomnography - fostering improved treatment and minimizing disturbances in the development of auditory function. We also recommend that this study be continued to increase patient numbers in each group.

## CONCLUSION

Based on the results of this study, it may be concluded that the presence of Obstruction Sleep Apnea/Hypopnea Syndrome correlates with auditory processing disturbances.
